# miR-132-3p and KLF7 as novel regulators of aortic stiffening-associated EndMT in type 2 diabetes mellitus

**DOI:** 10.1186/s13098-022-00966-y

**Published:** 2023-01-25

**Authors:** Melanie S. Hulshoff, Isabel N. Schellinger, Xingbo Xu, Jolien Fledderus, Sandip K. Rath, Fang Cheng Wong, Sabine Maamari, Josephina Haunschild, Guido Krenning, Uwe Raaz, Elisabeth M. Zeisberg

**Affiliations:** 1 Department of Cardiology and Pneumology, University Medical Center Göttingen, Georg-August-University, Robert-Koch-Str. 40, 37075 Göttingen, Germany; 2 German Center for Cardiovascular Research (DZHK), Partner Site, Göttingen, Germany; 3 Department of Pathology and Medical Biology, University Medical Center Groningen, University of Groningen, Hanzeplein 1, 9713 GZ Groningen, The Netherlands; 4 University Heart Center, Göttingen, Germany; 5 Department of Endocrinology, Nephrology and Rheumatology, University of Leipzig Medical Center, Leipzig, Germany; 6 University Department for Cardiac Surgery, Leipzig Heart Center, Leipzig, Germany

**Keywords:** Aortic stiffness, Endothelial-to-mesenchymal transition, KLF7, miR-132-3p, Type 2 diabetes

## Abstract

**Background:**

The prevalence of diabetes mellitus has risen considerably and currently affects more than 422 million people worldwide. Cardiovascular diseases including myocardial infarction and heart failure represent the major cause of death in type 2 diabetes (T2D). Diabetes patients exhibit accelerated aortic stiffening which is an independent predictor of cardiovascular disease and mortality. We recently showed that aortic stiffness precedes hypertension in a mouse model of diabetes (db/db mice), making aortic stiffness an early contributor to cardiovascular disease development. Elucidating how aortic stiffening develops is a pressing need in order to halt the pathophysiological process at an early time point.

**Methods:**

To assess EndMT occurrence, we performed co-immunofluorescence staining of an endothelial marker (CD31) with mesenchymal markers (α-SMA/S100A4) in aortic sections from db/db mice. Moreover, we performed qRT-PCR to analyze mRNA expression of EndMT transcription factors in aortic sections of db/db mice and diabetic patients. To identify the underlying mechanism by which EndMT contributes to aortic stiffening, we used aortas from db/db mice and diabetic patients in combination with high glucose-treated human umbilical vein endothelial cells (HUVECs) as an in vitro model of diabetes-associated EndMT.

**Results:**

We demonstrate robust CD31/α-SMA and CD31/S100A4 co-localization in aortic sections of db/db mice which was almost absent in control mice. Moreover, we demonstrate a significant upregulation of EndMT transcription factors in aortic sections of db/db mice and diabetic patients. As underlying regulator, we identified miR-132-3p as the most significantly downregulated miR in the micronome of db/db mice and high glucose-treated HUVECs. Indeed, miR-132-3p was also significantly downregulated in aortic tissue from diabetic patients. We identified Kruppel-like factor 7 (KLF7) as a target of miR-132-3p and show a significant upregulation of KLF7 in aortic sections of db/db mice and diabetic patients as well as in high glucose-treated HUVECs. We further demonstrate that miR-132-3p overexpression and KLF7 downregulation ameliorates EndMT in high glucose-treated HUVECs.

**Conclusions:**

We demonstrate for the first time that EndMT contributes to aortic stiffening in T2D. We identified miR-132-3p and KLF7 as novel EndMT regulators in this context. Altogether, this gives us new insights in the development of aortic stiffening in T2D.

**Supplementary Information:**

The online version contains supplementary material available at 10.1186/s13098-022-00966-y.

## Introduction

Throughout the world, diabetes is a growing health burden. Largely unknown in the early twentieth century, type 2 diabetes (T2D) is now the 7th leading cause of death in the USA mainly due to increased cardiovascular mortality in T2D [1, 2]. One mechanism linking diabetes to increased cardiovascular risk is accelerated arterial stiffening that is frequently observed in diabetic patients [3]. Stiffening of large arteries leads to various adverse hemodynamic consequences, rendering arterial stiffness an independent risk factor of cardiovascular disease [4, 5]. In that respect, the Hoorn study clearly demonstrated that T2D is especially associated with increased central arterial stiffness [6].

The aorta is the biggest vessel of the body and channels the blood flow from the heart to the periphery. Its elastic nature offers a buffering capacity (*Windkessel* function) to equalize blood flow during systole and diastole. As such, the aortic wall is an important responder to the biomechanical forces induced by the cyclic nature of blood flow.

Elevated arterial stiffness may result from increased collagen built-up in the arterial wall [7]. However, little is known about the underlying molecular mechanisms.

T2D is a chronic state characterized by hyperglycemic stimuli and low-grade inflammation [8]. Endothelial cells are early targets of these destructive conditions and hold a critical role in the production of extracellular matrix (ECM) proteins in diabetic complications [9–11]. Endothelial-to-mesenchymal transition (EndMT) is a biologic process that forces endothelial cells to undergo dynamic phenotypic switching (transition) in the context of sustained injury. Such changes in endothelial cells are manifested by a loss of endothelial markers and gain of mesenchymal markers [12–15]. EndMT has been shown to be a main source of fibroblasts triggering an increased production of ECM proteins in organ fibrosis [16–18].

New evidence suggests that EndMT is epigenetically regulated by a class of small non-coding RNAs called microRNA (miRs) [12, 19–22]. MiRs are short single stranded RNAs, that repress the expression of messenger RNAs (mRNAs) by binding to 3′UTR regions that are (partially) complimentary to their own code [23]. The number of mRNAs that may be affected by a single miR is estimated to be in the hundreds [24–27].

Here, we demonstrate that EndMT contributes to aortic stiffening in T2D and identify miR-132-3p and KLF7 as novel regulators in EndMT-triggered arterial stiffening.

## Methods

### Pressure myography

Pressure myography was performed to directly assess the passive aortic mechanics ex vivo as previously described (PubmedID 26208651). In brief, murine aortae were explanted, placed on specially designed stainless-steel cannulas and secured with silk surgical suture (10-0). The vessel was mounted in the heated chamber of a pressure arteriograph system (Model 110P, Danish Myotechnology, Copenhagen, Denmark) and stretched to in vivo length. Physiological saline solution (PSS) at 37 °C, aerated with 5% CO_2_/95% O_2_ was used to fill the vessel chamber and for aortic perfusion. After 3 preconditioning cycles, the aortic passive pressure-diameter relationship was determined by an automated protocol. The artery was pressurized from 0 to 180 mmHg in 18 mmHg increments, and the vessel’s outer diameter was simultaneously tracked by continuous computer video analysis (MyoVIEW software, Danish Myotechnology, Copenhagen, Denmark). The resting diameter d0 was quantified under 0 mmHg intraluminal (transmural) pressure and was not significantly different between the experimental groups tested (d0~1000 µm).

### Immunofluorescence staining of aortic sections of db/db mice

Male db/db mice (BKS.Cg-Dock7m+/+Leprdb/J) and their age-matched heterozygous non-diabetic controls (+/db) were purchased from the Jackson Laboratory (Bar Harbor, ME, USA). After 20 weeks, mice were exposed to inhalation of a lethal dose of isoflurane (concentration of 5%, Vet One, Meridian, ID, USA) in a closed chamber. Isoflurane was delivered from a vaporizer-system with O_2_ as a carrier. Thoracic aortas were harvested and snap-frozen in liquid nitrogen. All animal studies were reviewed and approved by the responsible local animal ethics review boards and procedures were conform to the guidelines from Directive 2010/63/EU of the European Parliament on the protection of animals used for scientific purposes. The cryo-sections were fixed with methanol at − 20 °C for 10 min and air-dried before washing three times with PBS. The sections were blocked using SEA BLOCK Blocking Buffer (Thermo Fisher Scientific, Waltham, MA, USA) and incubated at RT for 1.5 h. The primary antibody was added to the sections and incubated at 4 °C overnight. The next day, the slides were three times washed with PBS before adding the secondary antibody and incubating in the dark at RT for 45 min. The slides were washed three times with PBS and incubated with DAPI (1:1000 in PBS) at RT for 5 min. A final washing with PBS was performed before mounting the slides. The following primary antibodies and dilutions (in SEA BLOCK) were used: mouse anti-human CD31 (1:100, M0823, Dako, Carpinteria, CA, USA), rabbit anti-human S100A4 (1:50, A5114, Dako, Carpinteria, CA, USA), rabbit anti-mouse α-SMA (1:100, ab5694, Abcam, Cambridge, MA, USA) and rabbit anti-KLF7 (1:50, ab197690, Abcam, Cambridge, MA, USA). The secondary antibodies Alexa Fluor 647 goat-anti mouse (A21235, Invitrogen, Carlsbad, CA, USA) and Alexa Fluor 568 donkey anti-rabbit (A10042, Invitrogen, Carlsbad, CA, USA) were used in a 1:200 dilution. The stained slides were analysed using the Inverted Zeiss LSM 780 multiphoton laser scanning confocal microscope (Zeiss, Oberkochen, Germany).

### Human tissue sample acquisition and preparation

Human thoracic aortic samples from patients with diabetes (n = 5) and without diabetes (n = 5) who underwent open aortic surgery were collected during the procedure, snap-frozen and stored at − 80 °C. Groups were matched for age (patients with diabetes: 67.20 ± 2.059 years; patients without diabetes 65.60 ± 2.379 years; p = ns) and smoking status (patients with diabetes 40% smoker; patients without diabetes: 60% smoker; p = ns). Approval for studies on human tissue samples was obtained under informed consent and conducted in accordance with the Declaration of Helsinki.

### RNA extraction and quantitative real-time PCR for aortic sections of db/db mice and human tissue

Total RNA was extracted using TRIzol Reagent (Invitrogen, Carlsbad, CA, USA) and the PureLink RNA Mini Kit (Invitrogen, Carlsbad, CA, USA) according to the manufacturer’s protocol. The RNA was treated with Dnase I (Sigma-Aldrich, St. Louis, MO, USA) and the SuperScript II Reverse Transcriptase system (Invitrogen, Carlsbad, CA, USA) was used to synthesize cDNA according to the manufacturer’s protocol. For qRT-PCR, Fast SYBR Green Master Mix (Applied Biosystems, Foster City, CA, USA) was used in combination with the StepOne Plus Real-Time PCR system (Applied Biosystems, Foster City, CA, USA). The primers used for qRT-PCR are listed in Table 1. The relative expression levels were standardized to GAPDH using the ΔΔCt method.

**Table 1:** qRT-PCR primer sequences

Human name	Sequence	Mouse name	Sequence
GAPDH	F: GTGGACCTGACCTGCCGTCT R: GGAGGAGTGGGTGTCGCTGT	Gapdh	F: TGTAGACCATGTAGTTGAGGTCA R: AGGTCGGTGTGAACGGATTTG
SNAIL	F: GGCAATTTAACAATGTCTGAAAAGG R: GAATAGTTCTGGGAGACACATCG	Snail	F: GTGCCCACCTCCAAACCC R: AAGGACATGCGGGAGAAGG
SLUG	F: ACTCCGAAGCCAAATGACAA R: CTCTCTCTGTGGGTGTGTGT	Slug	F: CGCTCCTTCCTGGTCAAGA R: AGGTATAGGGTAACTTTCATAGAGATA
TWIST	F: GTCCGCAGTCTTACGAGGAG R: TGAATCTTGCTCAGCTTGTCC	Twist	F: TGATAGAAGTCTGAACACTCGTTTG R: GGCTGATTGGCAAGACCTCT
SMAD2	F: GGGTTTTGAAGCCGTCTATCAGC R: CCAACCACTGTAGAGGTCCATTC	Klf7	F: GGAAGGATGCGAGTGGCGTTTT R: CGCAAGATGGTCAGACCTGGAG
ZEB2	F: AATGCACAGAGTGTGGCAAGGC R: CTGCTGATGTGCGAACTGTAGG		
KLF7	F: CTCACGAGGCACTACAGGAAAC R: TGGCAACTCTGGCCTTTCGGTT		
PTEN	F: TGAGTTCCCTCAGCCGTTACCT R: GAGGTTTCCTCTGGTCCTGGTA		
ZBTB20	F: CTCTGCAACAAGACTTTCACCGC R: AGGAGAAGGAGCGCCAACAGAT		
DNMT3a	F: CCTCTTCGTTGGAGGAATGTGC R: GTTTCCGCACATGAGCACCTCA		

### Micronome data

The micronome of glucose-treated HUVECs and the micronome of aortic vascular smooth muscle cells from db/db mice and db/+controls are available at Gene Expression Omnibus (GEO), NCBI (GSE74296 and GSE74521).

### RNA quantification and qRT-PCR miR-132-3p in human subjects

Total microRNA was isolated using a TRIzol-based (Invitrogen, Carlsbad, CA, USA) RNA isolation protocol. Reverse transcription was performed by using the TaqMan microRNA Reverse Transcription kit (Applied Biosystems, Foster Biosystems, Foster City, USA) according to the manufacturer’s instructions. MicroRNA TaqMan assays (Applied Biosystems, Foster City, USA) for hsa-miR-132-3p was used. Amplification took place on a Fast-Real-Time PCR System (Applied Biosystems, Foster City, USA). All fold changes were calculated by the method of ΔΔCt.

### Cloning

A fragment of the human 3′UTR of KLF7 which contains two miR-132-3p binding sites was amplified using Phusion High-Fidelity DNA Polymerase (New England Biolabs, Ipswich, MA, USA) and the primers KLF7-3′UTR-F: 5′ AATTACTAGTACCATCCCTTCAAGACACGT; KLF7-3′UTR-R: 5′ AATTGTTTAAACCCCAGATCTTGAAGGTTGCTG. Both the amplified PCR product and the pMiR-REPORT miRNA expression vector (Invitrogen, Carlsbad, CA, USA) were digested using *SpeI* and *PmeI* restriction enzymes (New England Biolabs, Ipswich, MA, USA). Ligation was performed using the LigaFast Rapid DNA Ligation System (Promega, Madison, WI, USA) and transformed into One Shot TOP10 Chemically Competent *E. coli* (Invitrogen, Carlsbad, CA, USA). Miniprep was performed using the Zyppy Plasmid Miniprep Kit and the sequence of the insert was confirmed using sequencing. The generated pMiR-REPORT-KLF7-3′UTR plasmid was amplified using the HiSpeed Plasmid Midi Kit (Qiagen, Hilden, Germany).

### Cell culture and glucose treatment

Human embryonic kidney (HEK293) cells were cultured in DMEM medium (Gibco, Carlsbad, CA, USA) supplemented with 10% fetal bovine serum (Gibco, Carlsbad, CA, USA) and 1% penicillin–streptomycin (Gibco, Carlsbad, CA, USA). Human umbilical vein endothelial cells (HUVECs; Lonza, Basel, Switzerland) were cultured in Endothelial Cell Growth Medium (EGM-2MV, Lonza, Basel, Switzerland). For glucose treatment, 0.2 × 10^6^ cells were plated and cultured as previously described [29] to induce EndMT. 60 mM d-glucose was supplemented for 4 days and the medium was changed every 2 days. 5.5 mM d-glucose plus 54.5 mM d-mannitol was used as control.

### Transfection of HEK293 cells

0.5 × 10^6^ HEK293 cells were plated on a 6-well plate and incubated overnight. The next day, the HEK293 cells were transfected by using Lipofectamine 2000 (Thermo Fisher Scientific, Waltham, MA, USA). In short, 2 ug of pMiR-REPORT-KLF7-3′UTR and 1 ug of pGL4.73[*hRluc*/SV40] (renilla) vector (Promega, Madison, WI, USA) together with 5 nM of hsa-miR-132-3p miRCURY LNA miRNA mimic (YM00472088, Qiagen, Hilden, Germany) or negative control miRCURY LNA miRNA mimic (YM00479902, Qiagen, Hilden, Germany) was added in a tube containing 250 µL Opti-MEM Reduced Serum Medium (Gibco, Carlsbad, CA, USA). Another tube was prepared containing a mastermix of 250 µL Opti-MEM and 5 µL of Lipofectamine 2000 per transfection. The tubes were mixed at RT for 5 min before combining the Lipofectamine 2000 mixture with the DNA mixture. The tubes were mixed again before incubating at RT for 20 min. The transfection complexes were added drop-wise to the cells and incubated overnight. The next day, the medium was changed. The cells were collected 72 h after transfection.

### Luciferase reporter assay

The luciferase assay was performed using the Dual-Luciferase Reporter Assay System (Promega, Madison, WI, USA). In short, the cells were lysed with 1:5 diluted passive lysis buffer by shaking at RT for 30 min. Centrifugation at 13,000 rpm for 10 min was performed to collect the supernatant (containing the cell lysate). The cell lysate was added in triplicates to a 96 well plate before adding Luciferase Assay Reagent II (for Firefly luciferase activity) and Sto&Glo Reagent (for Renilla luciferase activity) to detect the luminescence with the help of a luminescence plate reader.

### Transfection of HUVECs

0.2 × 10^6^ HUVECs were plated on a 6-well plate and incubated overnight. The next day, the HUVECs were transfected by using Lipofectamine 2000 (Thermo Fisher Scientific, Waltham, MA, USA). In short, for miR-132-3p overexpression, 10 pmol of hsa-miR-132-3p miRCURY LNA miRNA mimic (YM00472088, Qiagen, Hilden, Germany) or Silencer Negative Control No.1 siRNA (AM4635, Ambion, Austin, TX, USA) was added in a tube containing 250 µL Opti-MEM (Gibco, Carlsbad, CA, USA). For KLF7 KO, 50 pmol of KLF7 Silencer Select siRNA (Ambion, Austin, TX, USA) or Silencer Negative Control No.1 siRNA (AM4635, Ambion, Austin, TX, USA) was added in a tube containing 250 µL Opti-MEM (Gibco, Carlsbad, CA, USA). Another tube was prepared containing a mix of 250 µL Opti-MEM and 5 µL of Lipofectamine 2000 per transfection. The tubes were incubated at RT for 5 min. before combining the Lipofectamine 2000 mixture with the siRNA/miRNA mixture. The Lipofectamine 2000 mix was combined with the siRNA/miRNA mix and incubated at RT for 20 min. The transfection complexes were added drop-wise to the cells and incubated for 4 h. After 4 h the medium was changed. The next day d-glucose and d-Mannitol treatment was started. The cells were collected 72 h after transfection.

### RNA extraction and qRT-PCR in HUVECs

Total RNA was extracted using TRIzol Reagent (Ambion, Austin, TX, USA) according to the manufacturer’s protocol. The RevertAid First Strand cDNA Synthesis Kit (Thermo Fisher Scientific, Waltham, MA, USA) was used to synthesize cDNA according to the manufacturer’s protocol. For qRT-PCR, Fast SYBR Green Master Mix (Applied Biosystems, Foster City, CA, USA) was used in combination with the Viia7 Real-Time PCR system (Applied Biosystems, Foster City, CA, USA). The primers used for qRT-PCR are listed in Table 1. The relative expression levels were standardized to GAPDH using the ΔΔCt method.

### RNA quantification and qRT-PCR miR-132-3p in HUVECs

Total microRNA was isolated using a TRIzol Reagent (Ambion, Austin, TX, USA) according to the manufacturer’s protocol. Reverse transcription was performed by using the TaqMan microRNA Reverse Transcription kit (Applied Biosystems, Foster City, CA, USA) and microRNA-specific stemloop primers (Table 2). For miR-qRT-PCR, analysis was performed on a mixture containing 5 ng cDNA equivalent, 0.1 µM sense primer, 0.1 µM antisense primer (Table 3) and Fast SYBR Green Master Mix (Applied Biosystems, Foster City, CA, USA). Analysis was performed on the Viia7 Real-Time PCR system (Applied Biosystems, Foster City, CA, USA). The relative expression levels were standardized to U6 using the ΔΔCt method.

**Table 2:** miRNA-specific CDNA synthesis primer sequences

Human name	Stem loop primer sequence
miR-132-3p-SL primer	GTCGTATCCAGTGCAGGGTCCGAGGTATTCGCACTGGATACGACCGACCATG
RNU6-SL primer	GTCGTATCCAGTGCAGGGTCCGAGGTATTCGCACTGGATACGACAAAAATATGG

**Table 3:** miR-132-3p qRT-PCR primer sequences

Human name	Sequence
miR-132-3p	F: TGCGGTAACAGTCTACAGC
RNU6	F: TGCGGCTGCGCAAGGATGA
miR-Reverse	R: GTGCAGGGTCCGAGGT

### Immunofluorescence staining of HUVECs

Cells were two times washed with PBS before being fixed with 2% PFA at RT for 30 min. After another two washes with PBS, cells were permeabilized with 0.5% Triton in PBS for 15 min. Cells were washed again 3× in PBS and blocked with blocking buffer (5% donkey or goat serum in PBS) for 10 min. Cells were incubated with primary antibody (1:100 in blocking buffer) overnight at 4 °C. The next day, cells were washed with PBS, PBS + 0.05% Tween-20 and again with PBS before incubation with secondary antibody (1:500) in DAPI (1:5000) and 2% Human Serum for 60 min at RT in the dark. Cells were washed with PBS, PBS + 0.05% Tween-20 and PBS and stored in PBS at 4 °C until analysis. The following primary antibodies were used: mouse anti-human CD31 (M0823, Dako, Santa Clara, CA, USA), rabbit anti-human SM22α (ab14106, Abcam, Cambridge, MA, USA). Alexa Fluor 647 donkey anti-mouse (A31571, Invitrogen, Carlsbad, CA, USA) and Alexa Fluor 568 goat anti-rabbit (A21069, Invitrogen, Carlsbad, CA, USA) were used as secondary antibodies. The cells were analyzed using the EVOS FL cell imaging system (Life Technologies, Carlsbad, CA, USA).

### Statistics

For pressure myography analysis, 2-way repeated measures ANOVA was used. Normality and homoscedasticity were tested to ensure that parametric testing was appropriate. The co-localization data is presented as the mean ± SEM. Co-localization was assessed in 3 aortic sections per mouse and average values of each mouse were calculated. The average values for each mouse (n = 4) was then used to calculate average per group. Statistical analysis was performed using a Student’s *t* test (two-tailed). For qPCR analysis, the data is presented as the mean ± SD. Statistical analysis was performed using 1-way ANOVA with Turkey multiple comparisons test. A value of p < 0.05 was considered statistically significant.

## Results

### Aortic stiffness is increased in diabetic mice

In a first step, we characterized the structural stiffness of the aortic wall in db/db mice and their +/db counterpart controls using ex vivo pressure myography. We found that the aortic segments taken from db/db mice exhibited significantly increased “material” stiffness of the explanted tissue (compared to +/db controls, Additional file 1: Figure S1).

### Aortas of db/db mice and diabetic patients, but not of control mice and subjects, display robust signs of endothelial-to-mesenchymal transition

**Fig. 1:**
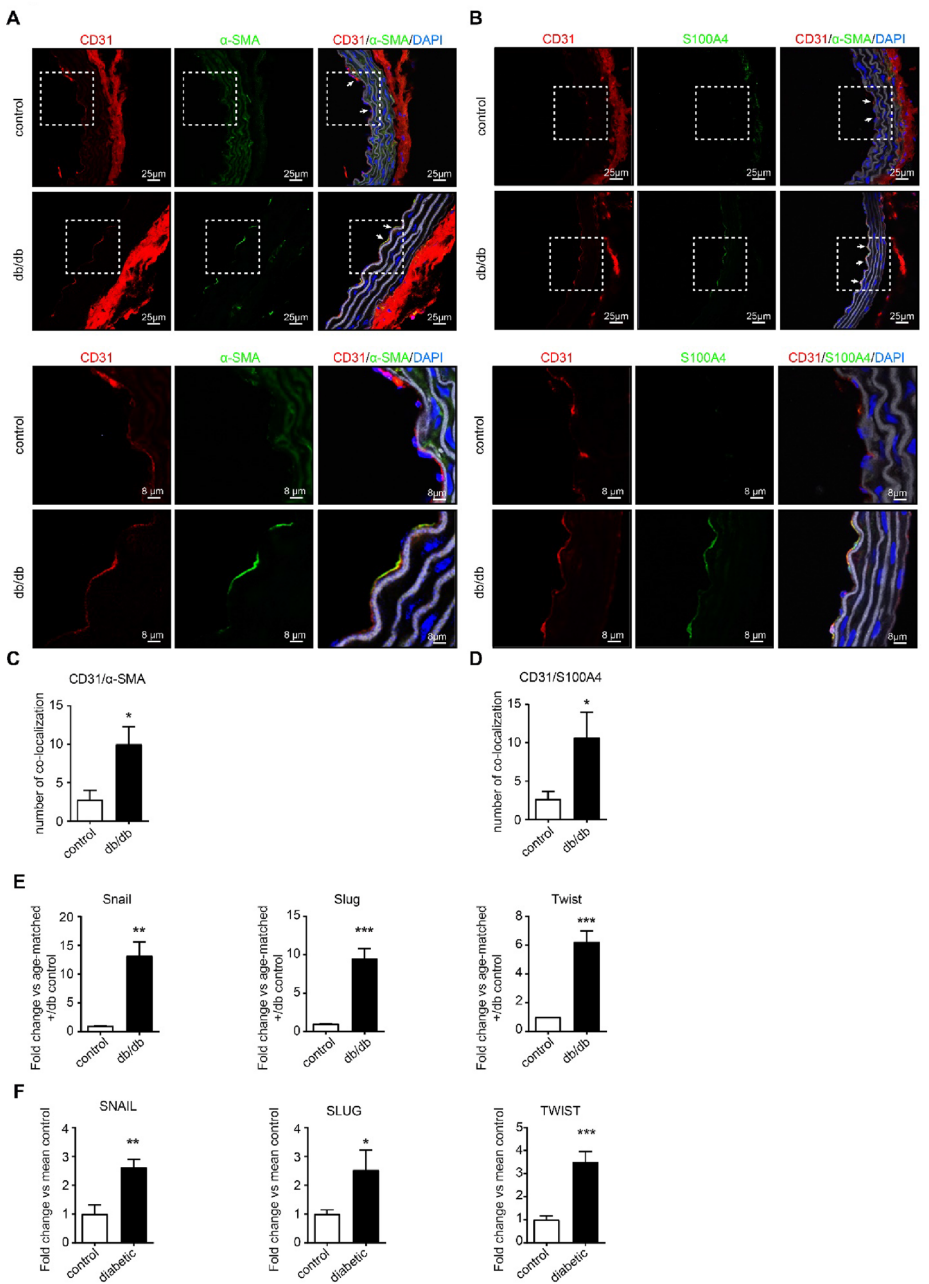
Endothelial-to-mesenchymal transition can be observed in aortas of db/db mice and diabetic patients. **A** Representative images (upper panel) of co-immunofluorescent staining of CD31 (red), α-SMA (green) and DAPI (blue). CD31 also stains the tunica adventitia. The elastic lamella is depicted in grey (autofluorescence). In db/db aorta, co-localization between CD31 and α-SMA is visible at the endothelial layer. Zoom-in (lower panel) of the tunica intima. CD31 marks the endothelial layer in control aorta (without α-SMA expression). In db/db aorta, robust CD31/α-SMA co-localization is present at the endothelial layer. **B** Representative images (upper panel) of co-immunofluorescent staining of CD31 (red), S100a4 (green) and DAPI (blue). CD31 also stains the tunica adventitia. The elastic lamella depicted in grey (autofluorescence). Co-localization of CD31 and S100a4 is present in db/db aorta (indicated by white arrows) and not in control aorta. Zoom-in (lower panel) of the tunica intima. In control aorta, CD31 marks the endothelial layer (without any S100a4 expression). Robust CD31/α-SMA co-localization is visible at the endothelial layer in db/db aorta. **C** Quantification of co-localization of CD31 with α-SMA upon co-immunostaining in aortas from db/db mice vs age-matched control mice. The amount of CD31/α-SMA co-localization is significantly higher in db/db aortas. **D** Quantification of CD31/S100A4 co-localization upon co-immunostaining in aortas from db/db mice vs age-matched control mice. The amount of CD31/S100A4 co-localization is significantly higher in db/db aortas. **E** qRT-PCR analysis of EndMT transcription factors Snail, Slug and Twist in db/db aortas vs aortas from age-matched control mice (n = 4). Snail, Slug and Twist are significantly upregulated in aortic tissue from db/db mice. **F** qRT-PCR analysis of EndMT transcription factors SNAIL, SLUG and TWIST in aortic tissue of diabetes patients vs control subjects (n = 7). SNAIL, SLUG and TWIST are significantly upregulated in aortic tissue of diabetic patients. Data is presented as mean value; error bars represent S.E.M; *p < 0.05; **p < 0.01; ***p < 0.001

To assess whether EndMT occurs during aortic stiffening, we performed confocal microscopy of co-immunofluorescent staining of the endothelial marker CD31 in combination with the mesenchymal markers α-SMA and S100A4 respectively in aortic sections of db/db mice (a murine model of T2D). We observed a robust co-localization of CD31 with both α-SMA and S100A4 in aortas of db/db mice which was almost absent in control (db/+) mice (Fig. 1A, B). The observed co-localization of CD31 with both α-SMA and S100A4 was significantly more pronounced in aortas of db/db mice when compared to aortas of control mice (for α-SMA 10 versus 2.8, p < 0.05, Fig. 1C; for S100A4 10.6 versus 1.6, p < 0.05, Fig. 1D). We then examined the mRNA expression of the EndMT transcription factors Snail, Slug and Twist in aortic tissue of db/db mice to further test the presence of EndMT. Indeed, Snail, Slug and Twist were significantly upregulated in aortic tissue from db/db mice when compared to control mice (Snail 13.5-fold, p < 0.01; Slug 9.4-fold, p < 0.001; Twist 6.1-fold, p < 0.001, Fig. 1E). To investigate if EndMT is also associated with aortic stiffening in patients with T2D, we also studied aortic tissue from patients with T2D and control subjects, and we found that *SNAIL*, *SLUG* and *TWIST* are significantly upregulated in T2D patients (*SNAIL* 2.6-fold, p < 0.01; *SLUG* 2.5-fold, p < 0.05; *TWIST* 3.4-fold, p < 0.001, Fig. 1F). Altogether, this demonstrates that EndMT can be observed in the context of T2D.

### miR-132-3p is downregulated in aortas of db/db mice and diabetic patients as well as in high glucose-induced EndMT

**Fig. 2:**
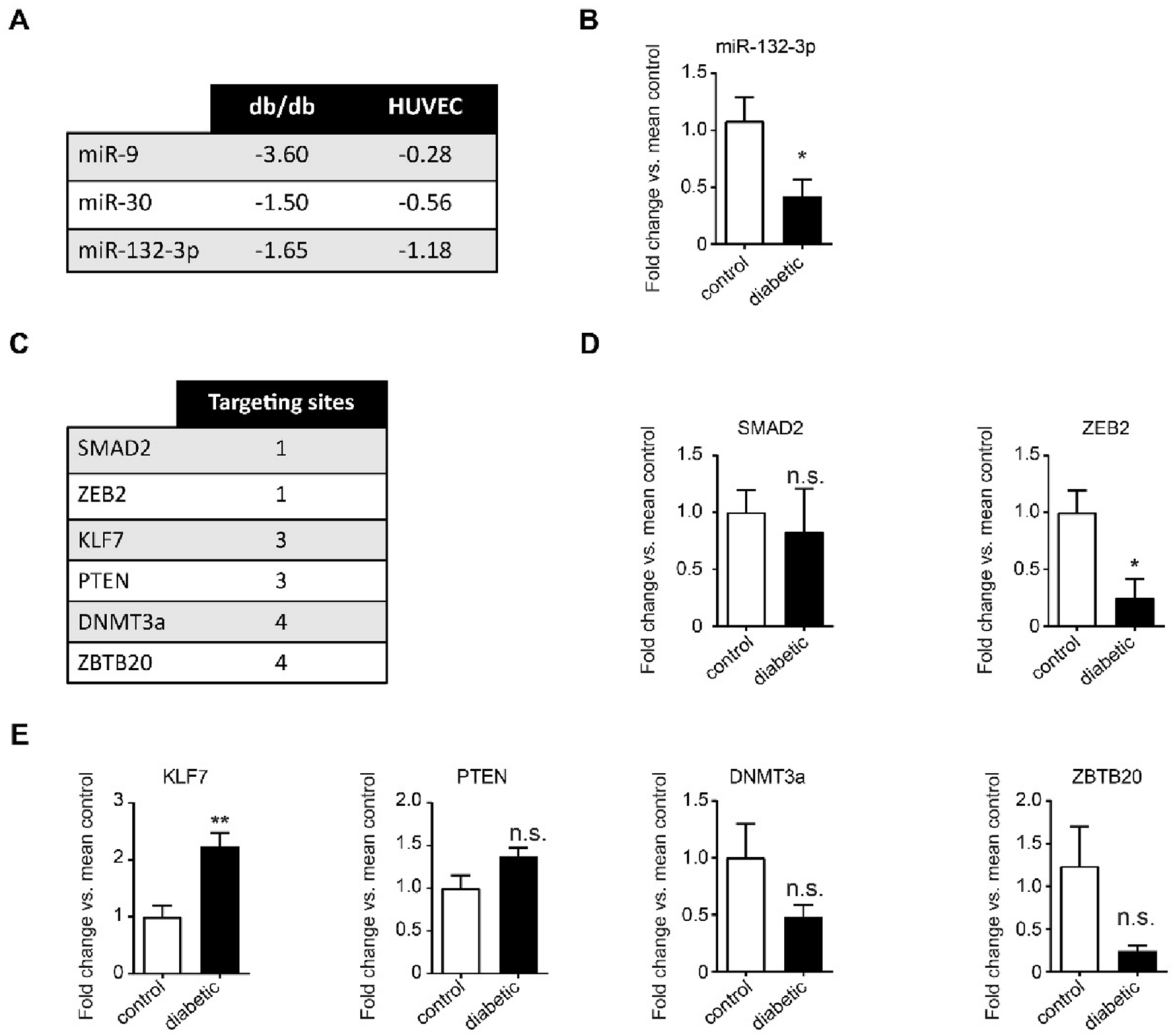
miR-132-3p is downregulated whereas KLF7 is upregulated in aortas of diabetic patients. **A** Overview of miRs that are downregulated in both the micronome of aortas from db/db mice and glucose-treated human umbilical vein endothelial cells (HUVECs). The values represent LogFC. **B** qRT-PCR analysis of miR-132-3p expression in aortic tissue from diabetic patients vs control subjects (n = 7). miR-132-3p is downregulated in aortic tissue from diabetic patients. **C** Overview of the predicted targets of miR-132-3p with the corresponding predicted targeting sites. **D** qRT-PCR analysis of SMAD2 and ZEB2 expression in diabetic patients vs control subjects (n = 7). SMAD2 remains unchanged and ZEB2 is downregulated in aortic tissue from diabetic patients. **E** qRT-PCR analysis of KLF7, PTEN, DNMT3a and ZBTB20 in aortic tissue from diabetic patients vs control subjects (n = 7). KLF7 is upregulated in aortic tissue from diabetic patients whereas PTEN, DNMT3a and ZBTB20 remain unchanged. All data is presented as mean value; error bars represent S.E.M.; n.s., not significant; *p < 0.05; **p < 0.01

To identify the underlying mechanism by which EndMT is regulated in the context of T2D, we overlapped the micronome of aortas from db/db mice with the micronome of high glucose-treated human umbilical vein endothelial cells (HUVECs) (available at Gene Expression Omnibus (GEO), NCBI, GSE74296 and GSE74521). Three microRNAs (miRs) were significantly downregulated in both datasets: miR-9, miR-30 and miR-132-3p (Fig. 2A). Since miR-132-3p was the highest downregulated in glucose-treated HUVECs and the second highest downregulated in aortas of db/db mice, we decided to further examine the expression of this miR in aortic tissue of T2D patients. Indeed, we observed a significant downregulation of miR-132-3p in aortic sections of T2D patients when compared to control subjects (2.4-fold, p < 0.05, Fig. 2B). Since miR-132-3p is universally downregulated in high glucose-treated HUVECs and in aortic tissue of db/db mice and diabetic patients, we identified miR-132-3p as possible regulator of EndMT-triggered aortic stiffening in T2D.

### miR-132-3p predicted target KLF7 is upregulated in aortas of diabetic patients

To further investigate the role of miR-132-3p in regulating EndMT, we identified possible targets of miR-132-3p in humans through the online prediction tool TargetScan. This revealed two well-known EndMT regulators: SMAD2, which facilitates TGF-β signaling, and ZEB2, a transcription factor regulating epithelial to mesenchymal transition (EMT) (Fig. 2C). SMAD2 mRNA expression was unaltered in aortas of T2D patients when compared to control subjects whereas ZEB2 was significantly downregulated (SMAD2 not significant; ZEB2, 4.1-fold, p < 0.05, Fig. 2D). This suggests that these two predicted targets of miR-132-3p do not play a role in this context. We next examined the mRNA expression of those predicted miR-132-3p targets with the most putative binding sites for miR-132-3p in their 3′UTR: KLF7, PTEN, DNMT3a and ZBTB20 (Fig. 2C). Out of these four predicted targets, only KLF7 is significantly upregulated in aortas of diabetes patients whereas DNMT3a, ZBTB20 and PTEN remain unaltered when compared to control subjects (KLF7 2.2-fold, p < 0.01; DNMT3/ZBTB20/PTEN not significant, Fig. 2E). This suggests that miR-132-3p targets KLF7 during aortic stiffening in diabetic patients.

### KLF7 is upregulated during EndMT-triggered aortic stiffening in db/db mice

**Fig. 3:**
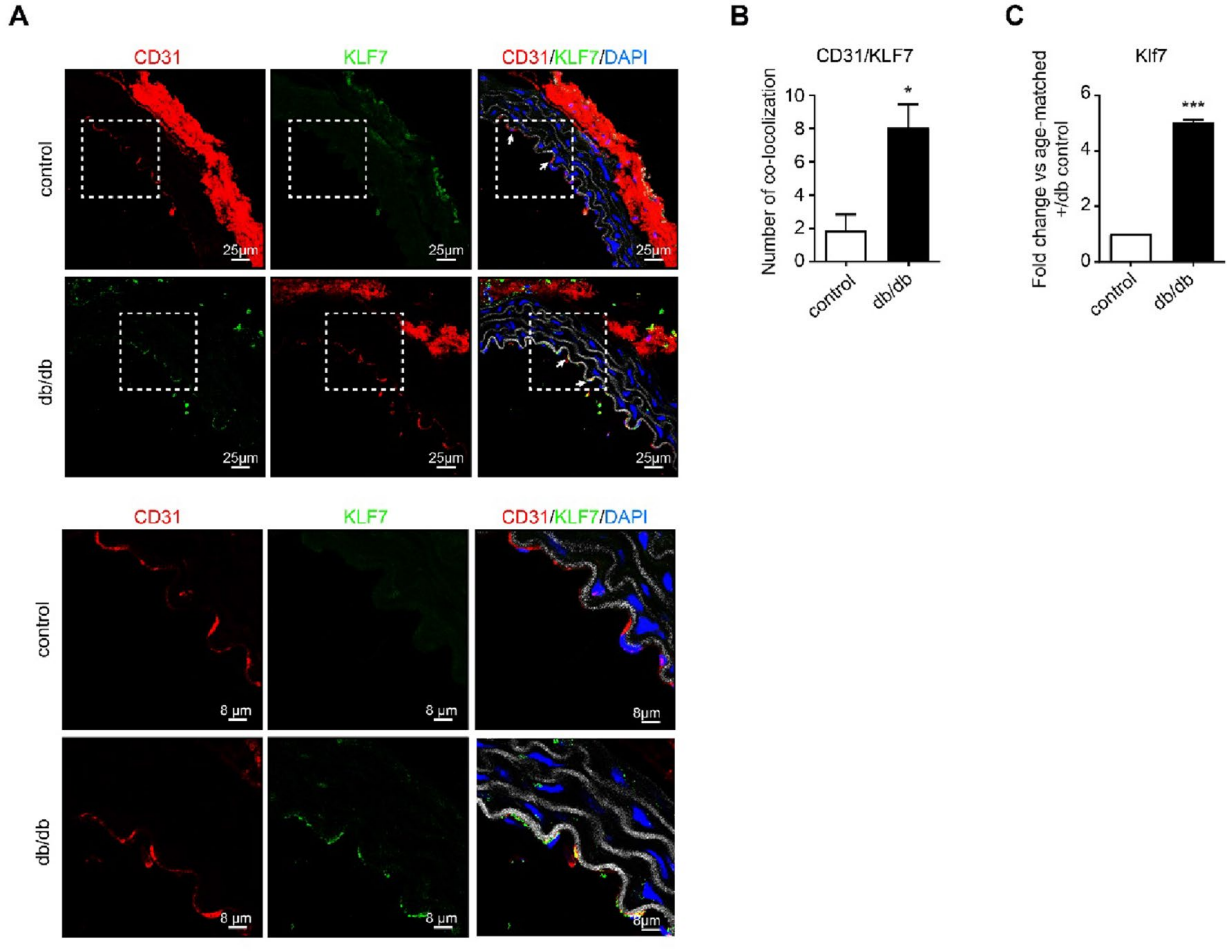
KLF7 is upregulated during EndMT-triggered aortic stiffening in db/db mice. **A** Representative images (upper panel) of co-immunofluorescent staining of CD31 (in red), KLF7 (in green) and DAPI (in blue). The elastic lamella is depicted in grey (autofluorescence). In the db/db aorta, co-localization between CD31 and KLF7 is visible at the endothelial layer (indicated by white arrows). Zoom-in (lower panel) of the tunica intima. CD31 marks the endothelial layer in the control aorta (without KLF7 expression). In the db/db aorta, robust co-localization between CD31 and KLF7 is present at the endothelial layer. **B** Quantification of co-localization of CD31 with KLF7 upon co-immunostaining in aortas from db/db mice vs age-matched control mice. The amount of co-localization of CD31 with KLF7 is significantly higher in aortas from db/db mice. **C** qRT-PCR analysis of KLF7 in aortas of db/db mice (n = 4) vs control. KLF7 is upregulated in aortic tissue of db/db mice. The data is presented as mean value; error bars represent S.E.M; *p < 0.05; ***p < 0.001

To assess whether KLF7 plays a role during EndMT-triggered aortic stiffness, we performed co-immunofluorescent staining of CD31 in combination with KLF7 in aortic sections of db/db mice. With confocal microscopy, we observed a robust co-localization of CD31 with KLF7 in aortas of db/db mice which was almost completely absent in control mice (Fig. 3A). The observed co-localization of CD31 with KLF7 was significantly more in aortas of db/db mice when compared to aortas of control mice (4.2-fold, p < 0.05, Fig. 3B). Finally, we examined the mRNA expression of KLF7 in aortic tissue of db/db mice. We also observed a significant upregulation of KLF7 in aortas of db/db mice when compared to control mice (4.9-fold, p < 0.001, Fig. 3C). This suggests that KLF7 plays a role in EndMT-triggered aortic stiffness in T2D.

### miR-132-3p overexpression ameliorates high glucose-induced EndMT

**Fig. 4:**
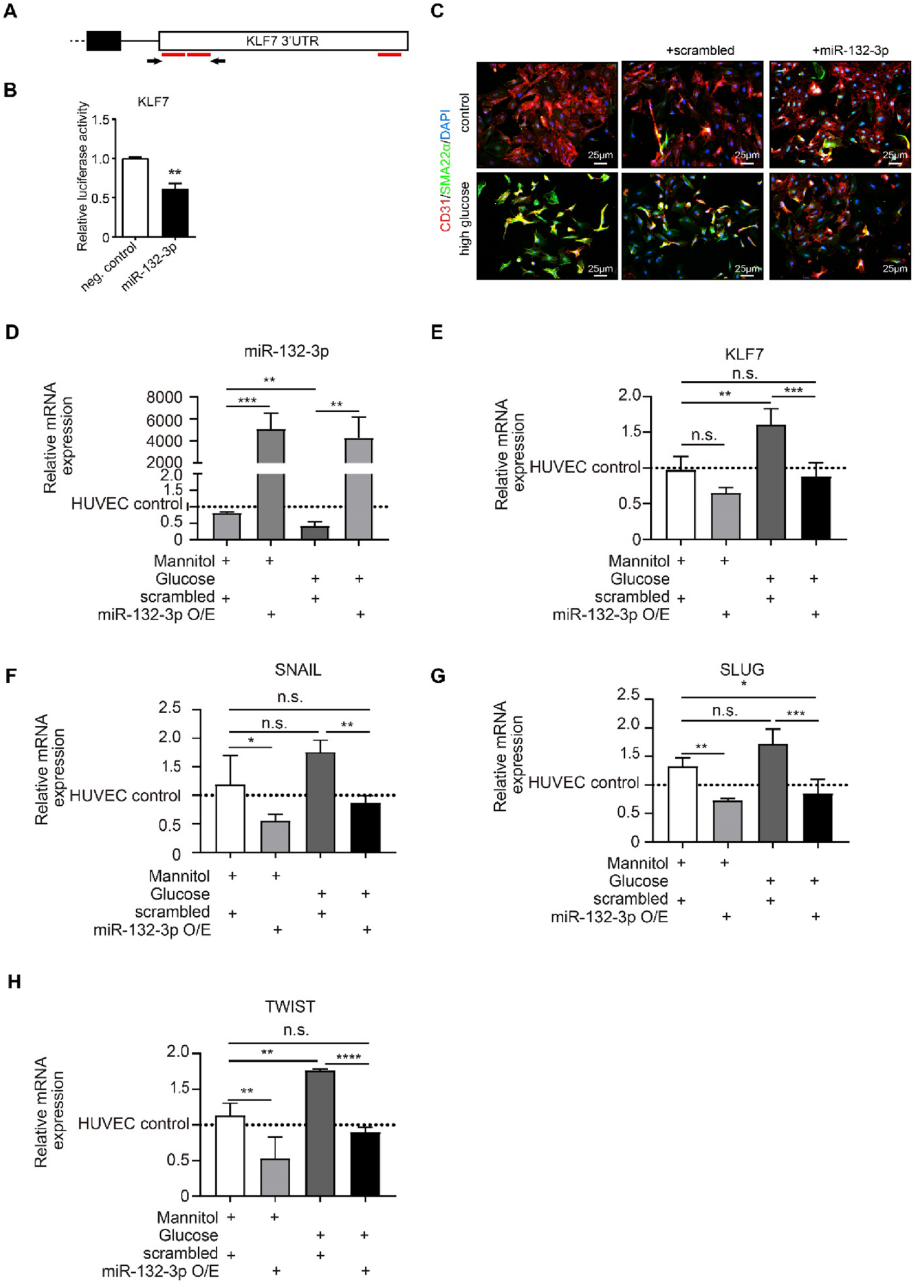
miR-132-3p targets KLF7 and overexpression of miR-132-3p ameliorates high glucose-induced EndMT. **A** Schematic demonstrating the 3′UTR of KLF7 where the predicted miR-132-3p target sites are depicted in red. The arrows represent the primers that were used to clone pMiR-REPORT-KLF7-3′UTR. **B** Luciferase assay of HEK293 cells transfected with pMiR-REPORT-KLF7-3′UTR in combination with a pGL4.73[hRluc/SV40] (renilla) vector with either a miR-132-3p mimic or negative control. The relative luciferase activity of the KLF7 3′UTR is downregulated upon co-transfection with the miR-132-3p mimic. **C** Co-immunostaining of CD31 with SM22α in high glucose-treated human umbilical vein endothelial cells (HUVECs) vs control. High glucose treatment results in loss of CD31 and cobblestone morphology and gain of SM22α accompanied by a spindle-shaped morphology. Overexpression of miR-132-3p in combination with high glucose treatment results in loss of SM22α expression and spindle-shaped morphology, and increase in CD31 expression and coble-stone morphology when compared to transfection with scrambled in combination with high glucose treatment. **D** qRT-PCR analysis of miR-132-3p in high glucose and mannitol conditions in combination with overexpression of miR-132-3p or scrambled. miR-132-3p is significantly downregulated in high glucose-treated HUVECs. In addition, miR-132-3p is significantly upregulated upon overexpression of miR-132-3p in both high glucose and mannitol conditions. **E–G** qRT-PCR analysis of KLF7 and the EndMT transcription factors SNAIL, SLUG and TWIST in high glucose (HG)-treated HUVECs vs control. KLF7, SNAIL, SLUG and TWIST are significantly upregulated in high glucose-treated HUVECs whereas this effect is abolished upon overexpression with miR-132-3p in high glucose conditions. Overexpression of miR-132-3p also decreases the expression of KLF7, SNAIL, SLUG and TWIST in mannitol conditions. The data is presented as mean value; error bars represent S.E.M.; n = 4 independent biological replicates; *p < 0.05; **p < 0.01; ***p < 0.001

To confirm that miR-132-3p targets KLF7, we cloned a fragment of the human 3′UTR of KLF7 in a pMiR-REPORT luciferase vector and transfected HEK293 cells with both the pMiR-REPORT-KLF7-3′UTR and a miR-132-3p mimic or negative control (Fig. 4A). We show that co-transfection with the miR-132-3p mimic results in a significant reduction of the luciferase activity of the KLF7 3′UTR when compared to the negative control, demonstrating that miR-132-3p indeed targets KLF7 (1.6-fold, p < 0.01, Fig. 4B).

To further explore the role of miR-132-3p in high glucose-induced EndMT, we overexpressed miR-132-3p in high glucose-treated HUVECs. Co-immunostaining of CD31 in combination with SM22α showed that high glucose treatment results in a decrease in the expression of CD31 and cobblestone morphology and an increase in SM22α expression accompanied with a spindle-shaped morphology, all indicative of EndMT (Fig. 4C). Moreover, qRT-PCR analysis revealed upregulation of KLF7, SNAIL, SLUG and TWIST upon high glucose treatment, and downregulation of miR-132-3p upon high glucose treatment, confirming the presence of EndMT, the downregulation of miR-132-3p and the upregulation of KLF7 in high glucose-induced EndMT (miR-132-3p 1.9-fold, p < 0.01; KLF7 1.6-fold, p < 0.01; SNAIL 1.75-fold, p = 0.06; SLUG 1.71-fold, p = 0.06; TWIST 1.75-fold, p < 0.01, Fig. 4D–H). After having confirmed the overexpression of miR-132-3p (in mannitol conditions 5083-fold, p < 0.0005; in high glucose conditions 4279-fold, p < 0.005, Fig. 4D), we demonstrated that overexpression of miR-132-3p in combination with high glucose treatment ameliorates high glucose-induced EndMT as shown by an increase in CD31 expression and of cobblestone morphology, and a decrease in SM22α expression and of spindle-shaped morphology (Fig. 4C). Moreover, overexpression of miR-132-3p decreases the expression of KLF7, SNAIL, SLUG, and TWIST in both high glucose (KLF7 1.82-fold, p < 0.0005; SNAIL 2.03-fold, p < 0.01; SLUG 2.03-fold, p < 0.0001; TWIST 1.95-fold, p < 0.001, Fig. 4E–H) and mannitol conditions (KLF7 1.56-fold, p < 0.05; SNAIL 1.81-fold, p < 0.05; SLUG 1.38-fold, p < 0.01; TWIST 1.92-fold, p < 0.01, Fig. 4E–H). This indicates that miR-132-3p overexpression ameliorates high glucose-induced EndMT in vitro.

### KLF7 downregulation ameliorates high-glucose EndMT

**Fig. 5:**
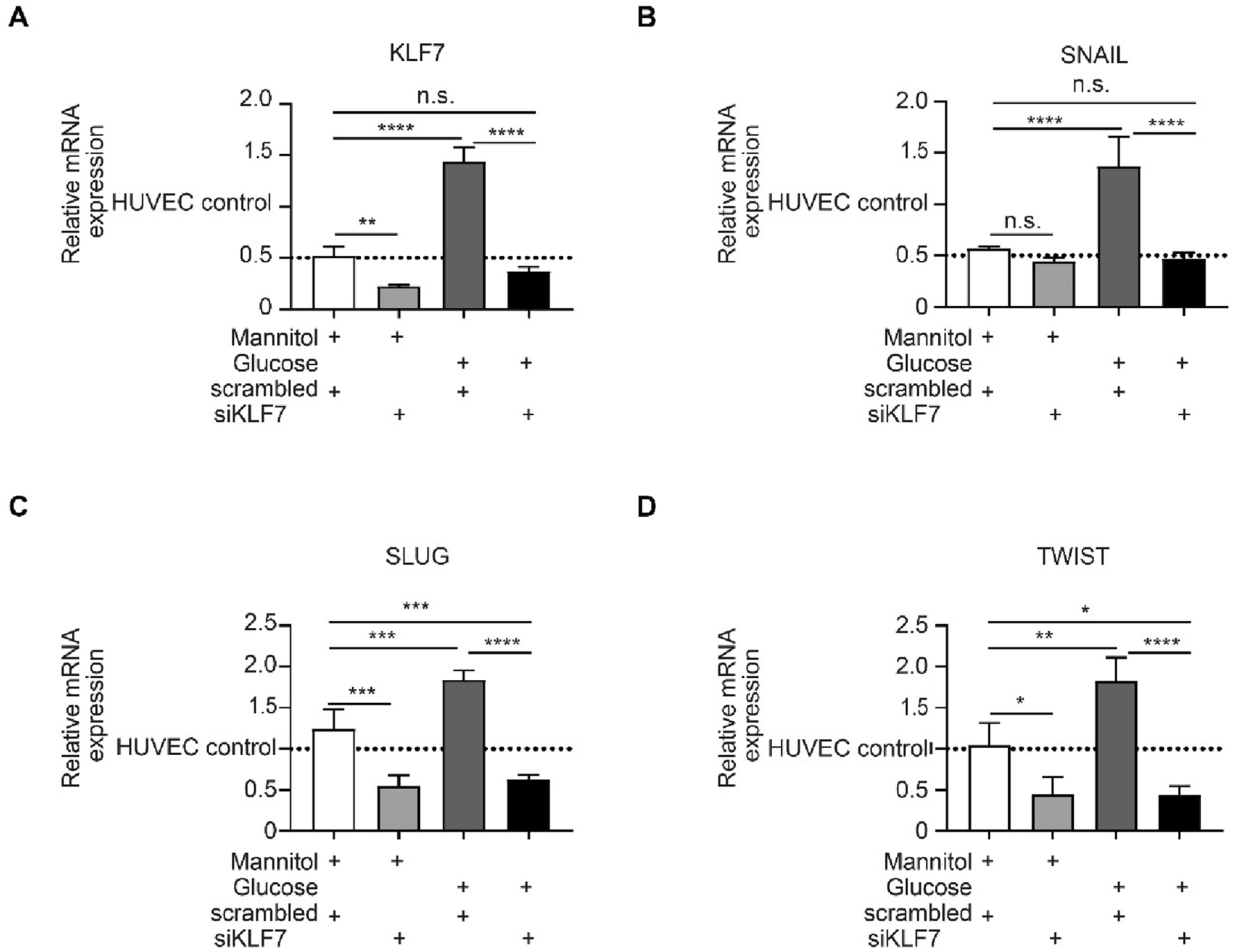
Knockdown of KFL7 ameliorates high glucose-induced EndMT. **A–D** qRT-PCR analysis of KLF7 and the EndMT transcription factors SNAIL, SLUG and TWIST in high glucose (HG)-treated HUVECs vs control. KLF7, SNAIL, SLUG and TWIST are significantly upregulated in high glucose-treated HUVECs whereas this effect is abolished upon knockdown of KLF7 in high glucose conditions. Knockdown of KLF7 also decreases the expression of KLF7, SLUG and TWIST in mannitol conditions. The data is presented as mean value; error bars represent S.E.M.; n = 4 independent biological replicates; *p < 0.05; **p < 0.01; ***p < 0.001

After having established the role of miR-132-3p on high glucose-induced EndMT, we further characterized the role of KLF7 on high glucose-induced EndMT. Therefore, we downregulated KLF7 in high glucose-treated HUVECs. Downregulation of KLF7 decreases the expression of KLF7, SNAIL, SLUG, and TWIST in both high glucose (KLF7 3.86-fold, p < 0.0001; SNAIL 2.9-fold, p < 0.0001; SLUG 2.9-fold, p < 0.0001; TWIST 4.23-fold, p < 0.001, Fig. 5A–D) and KLF7, SLUG, and TWIST in mannitol conditions (KLF7 2.27-fold, p < 0.01; SLUG 1.85-fold, p < 0.001; TWIST 2.27-fold, p < 0.05, Fig. 5A–D).

**Fig. 6:**
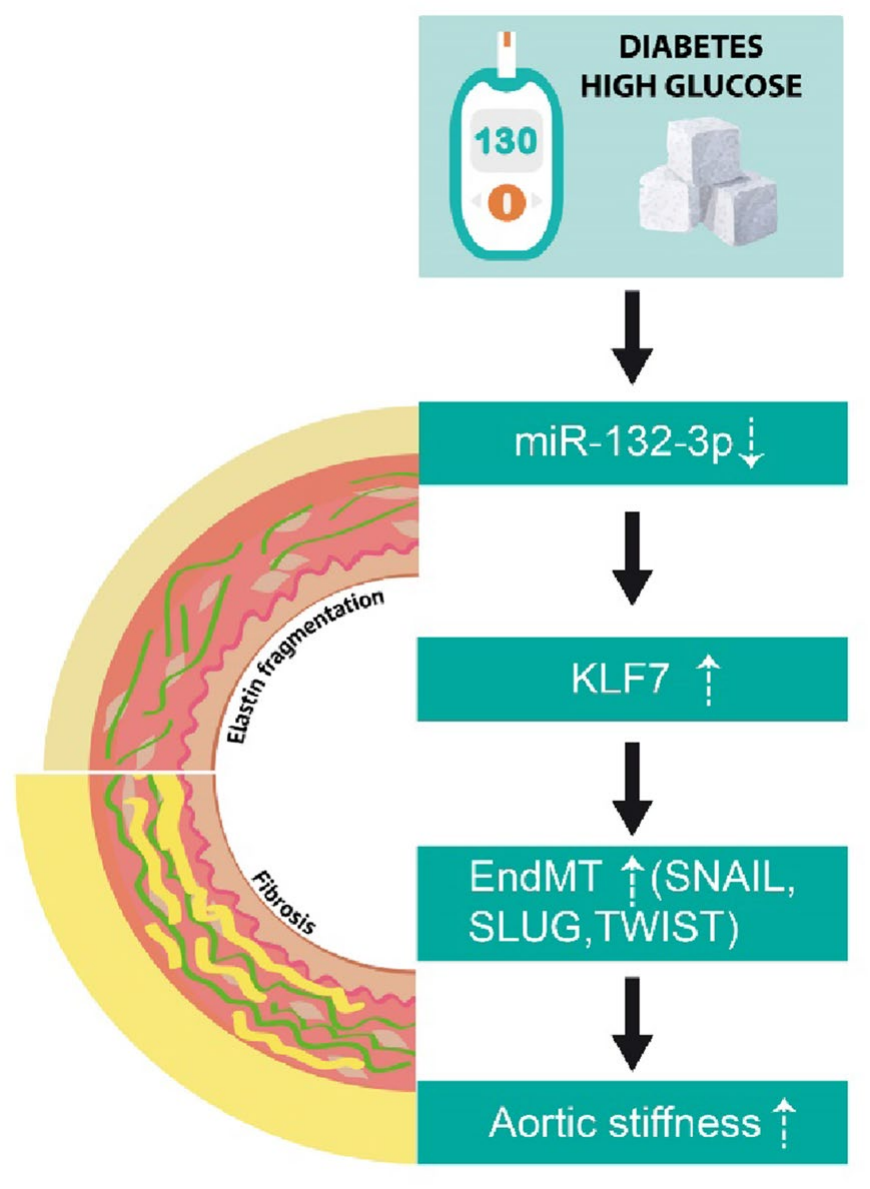
Proposed mechanism triggering EndMT in diabetes-related aortic stiffening. Under diabetic or high glucose conditions, miR-132-3p is downregulated which activates the expression of its target KLF7. KLF7 is upregulated in aortas of both diabetic patients and an in vivo model of diabetes, as well as in high glucose-treated endothelial cells. Moreover, KLF7 co-localizes with the endothelial marker CD31 in diabetic mice, which suggests its contribution to EndMT in diabetes-related aortic stiffening

Altogether, this suggests that KLF7 downregulation ameliorates high glucose-induced EndMT in vitro and that miR-132-3p regulates KLF7 in EndMT-triggered diabetes-related aortic stiffening (Fig. 6).

## Discussion

In this study, we demonstrate that aortas of db/db mice (a murine model of type 2 diabetes) and diabetes patients but not of control mice and subjects display robust signs of EndMT. We identified that miR-132-3p is downregulated in aortas of both db/db mice and diabetic patients as well as in an in vitro model of glucose-induced EndMT. Moreover, we demonstrate that miR-132-3p targets KLF7, which is upregulated in an in vitro model of diabetes-associated EndMT, during EndMT-triggered aortic stiffening in db/db mice as well as in aortas of diabetes patients. Finally, we show that miR-132-3p overexpression as well as KLF7 downregulation ameliorates EndMT in an in vitro model of diabetes-associated EndMT, thereby identifying both miR-132-3p and KLF7 as novel regulators of aortic stiffening-associated EndMT in type 2 diabetes mellitus. A summary of the proposed mechanism is provided in Fig. 6.

Accelerated aortic stiffening is an independent predictor of cardiovascular disease and mortality in diabetes patients. We previously showed that aortic stiffness precedes hypertension in db/db mice, making aortic stiffness an early contributor to cardiovascular disease development [7]. Elucidating how aortic stiffening develops is therefore a pressing need in order to halt the pathophysiological process at an early time point. We now identify EndMT as a novel contributor to aortic stiffness in T2D. This is consistent with the literature in which high glucose-induced EndMT has been demonstrated in vitro before [28, 29], and has also been reported to be associated with diabetic cardiomyopathy [30–33].

MicroRNAs have been shown to regulate EndMT in the context of diabetes [20, 34, 35]. We are the first to identify miR-132-3p as a possible regulator of EndMT and its association with both cardiovascular disease and T2D. Other studies have shown that downregulation of miR-132-3p promotes migration and proliferation in the context of carcinoma, which suggests a role for miR-132-3p in epithelial-to-mesenchymal transition (EMT), a cellular transition process similar to EndMT [36–40].

We demonstrate that miR-132-3p regulates KLF7 and thereby identify KLF7 as a novel regulator of EndMT. KLF7 is a member of the KLF7 family of zinc finger transcription factors which are known to play an important role in development and cellular differentiation processes such as EMT [41–44]. Moreover, KLF7 has been identified as one of the core transcription factors that regulate coronary artery disease-associated pathways [45]. This supports our data, as it has been shown that EndMT drives the progression of coronary artery disease [14, 46]. This confirms that KLF7 might be an important player in EndMT-triggered arterial stiffness in T2D.

To conclude, we demonstrate that EndMT contributes to aortic stiffening in T2D. We also identified miR-132-3p and KLF7 as potential regulators of EndMT in this context. Altogether, this provides new insights in the development of aortic stiffening in T2D.

## Supplementary Information

**Additional file 1: Fig. S1.** db/db mice exhibit increased structural aortic stiffness. Aortic pressure diameter curves from db/db mice vs. +/db controls. *p < 0.05 vs. +/db controls. (n = 5/group)

## Data Availability

The datasets supporting the conclusions of this article are available at Gene Expression Omnibus (GEO), NCBI, GSE74296 and GSE74521. Hyperlink to dataset GSE74296: https://www.ncbi.nlm.nih.gov/geo/query/acc.cgi?acc=GSE74296 Hyperlink to dataset GSE74521: https://www.ncbi.nlm.nih.gov/geo/query/acc.cgi?acc=GSE74521

## Abbreviations

ECM: Extracellular matrix
EndMT: Endothelial-to-mesenchymal transition
HUVEC: Human umbilical vein endothelial cells
KLF7: Kruppel-like factor 7
mRNAs: Messenger RNAs
miRs: MicroRNA
T2D: Type 2 diabetes
