# Supplementary material for: miR-132-3p and KLF7 as novel regulators of aortic stiffening-associated EndMT in type 2 diabetes mellitus
Source: Diabetol Metab Syndr. 2023 Jan 25;15:11. doi: 10.1186/s13098-022-00966-y (PMC9875453; doi:10.1186/s13098-022-00966-y)
Supplement: Supplementary file 1 — Additional file 1: Fig. S1. db/db mice exhibit increased structural aortic stiffness. Aortic pressure diameter curves from db/db mice vs. +/db controls. *p < 0.05 vs. +/db controls. (n = 5/group) [file 13098_2022_966_MOESM1_ESM.docx]

**
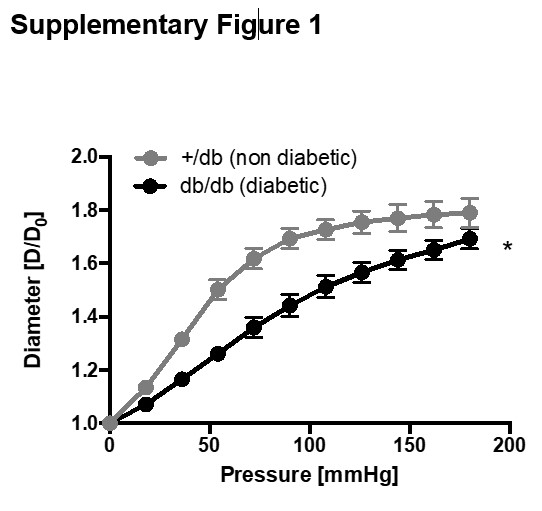
**

**Suppl.Fig.1. db/db mice exhibit increased structural aortic stiffness**

Aortic pressure diameter curves from db/db mice vs. +/db controls. * p<0.05 vs. +/db controls. (n=5/group).
